# Acute nontraumatic torticollis in a patient with right lower quadrant pain: case report

**DOI:** 10.11604/pamj.2015.21.218.5984

**Published:** 2015-07-27

**Authors:** Faik Yaylak, Sezgin Zeren, Zülfü Bayhan, Refik Bademci, Cigdem Devir

**Affiliations:** 1 Dumlupinar University, Faculty of Medicine, Department of General Surgery, Kutahya, Turkey; 2 Istanbul Bilim University, Faculty of Medicine, Department of General Surgery, Istanbul, Turkey; 3 Evliya Celebi Research and Education Hospital, Department of Radiology, Kutahya, Turkey

**Keywords:** Right lower quadrant pain, acute appendicitis, preoperative evaluation, torticollis

## Abstract

Right lower quadrant pain is one of the most common symptoms of the emergency patients. For accurate diagnosis and treatment; the patients must be questioned and examined very well. Also accompanying conditions due to right lower quadrant pain may be noticed. In this case presentation, we discussed a patient who was presented with right lower quadrant pain and cervical dystonia. By limiting the usage of metoclopramide the patient was followed seamlessly. In this case presentation we want to accentuate that a patient who with abdominal pain may be presented with rare symptoms such of dystonia. In such conditions a detailed anamnesis and physical examination are the first steps of the evaluation to prevent potential hazardous outcomes. In particular, a surgeon must be always carefully while taking history and examining the patient.

## Introduction

Acute right lower abdominal pain is a common surgical presentation due to acute appendicitis and merits a thorough clinical evaluation and preoperative assessment is some patients. These procedures should be directed to obtain a conclusive preoperative diagnosis, appropriate preparation for anesthesia and surgery. This approach should not be overwhelmed not only to minimize potential complication in individual patients to prevent medico legal interests and risks but to minimize morbidity and mortality rates. Previously acute dystonic reactions have been presented in some patients with right lower quadrant pain due to medications [1]. In addition, non traumatic torticollis due to nontraumatic atlantoaxial subluxation has been reported after surgery [2]. Thus, such conditions should be recognized and timely managed.

## Patient and observation

A 18 years old boy was presented to our clinic with acute lower upper abdominal and back pain from emergency service. In detailed questioning he has confirmed a recentin crease in stool frequency without mucus and blood. His previous medical and surgical history was not significant. He denied a specific medical condition in his family. He was a non-smoker and a non-alcohol drinker. Usage of neuroleptics (antipsychotics such as haloperidol), other medications (such as tricyclic anti-depressants, or anticonvulsants) and herbals were questioned and all were refused by the patient. However, emergency service records have documented the usage of anti-emetics (metoclopramide 10 mg, im) and other medications (ranitidin 50 mg iv and Hiyosin-N-butilbromür 20 mg im) two hours prior to developement of abnormal cervical posture and muscular spasm on the left arm. Physical examination revealed acute dystonic reaction with right sided torticollis and minimal oculogyric crisis (upward and out ward turning of the eyes) were observed. These findings were observed to resolve after a rest of one hour in the bed in the Standard hospital room.

## Discussion

**Figure 1:**
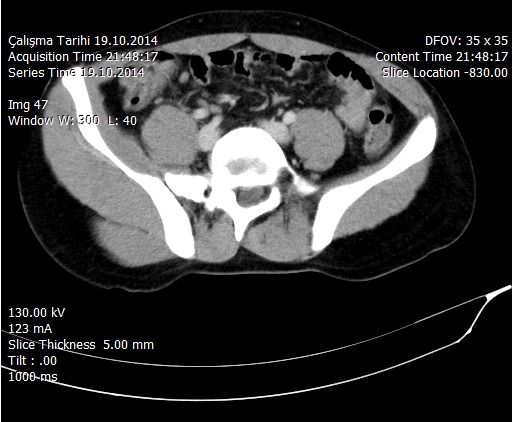
Distinctive pancreatic tissue in intravenous contrast enhanced abdominopelvic computerized tomography

**Figure 2:**
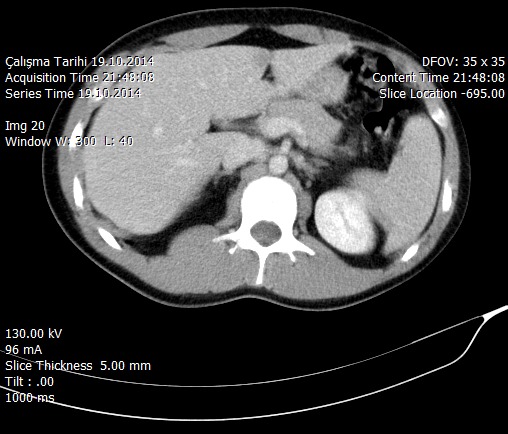
Mesenteric lenfadenitis in right lower quadrant of the abdomen in intravenous contrast enhanced abdominopelvic computerized tomography

The primary intention of this patient was to exclude to need of an emergent surgery for appendicitis. However, due to overall clinical presentation in this patient with back pain, elevated leukocytes, c-reactive peptide, clusters of lymphadenopathy and acute dystonic reactions have directed us to observe patient. Metoclopramide usage was restricted and the patient was consultated with infectious disease specialist and neurologists. Advised and required tests were performed to exclude the disease. During hospitalization period metoclopramide usage was restricted to prevent an induction of acute dystonic reaction recurrence. With serial physical examination and review of the imaging findings (Figure 1, Figure 2), we excluded a need for emergent surgery. Nontraumatic atlantoaxial subluxation has been reported due an infection or an inflammation at the head and neck region and after surgery [3]. Early diagnosis and treatment is essential to prevent neurological sequelae and/or painful and lasting deformity of the neck pediatric age group and any upper respiratory tract infection are risk factors. However, classical signs such as neck stiffness and torticollis-associated painful neck movements were absent in our patient. Acute dystonic reactions have been reported in a previous report by Oyewole et al. From Nigeria in a single 28-year-old female undergraduate who presented to the medical unit with 4 days history of acute lower abdominal pains, high-grade intermittent fever, and persistent vomiting [1]. Our patient's clinical presentation was comparable with their report. In our case torticollis and upward or outward turning of the eyes were observed. On the other hand other dystonic reactions were not observed. All clinical findings were resolved and recurrence was not observed. Pathophysiology of acute dystonic reactions is not clear. A central dopamine transmission deficit is believed to result with overactive striatal acetylcholine release. Anti-cholinergic medications may reverse this over active release [4]. However, clinical suspicion and early diagnosis is essential to prevent fatal outcomes. Here it is the surgeons’ role in such conditions to adequately evaluate the patient and whenever possible prevent the triggering factors such as electrolyte imbalances or usage of anti-emetics prior to neurology consultation. We strictly recommend a consultation of the patient to a neuroimmunologist if it is possible. Previously the authors have reported unusual findings in the appendectomized patients [5]. Together with this case, were commend multidisciplinary approach to patient with right lower quadrant pain with suspicious or unusual presentations. Our case presentation emphasize the significance of evaluating the patient systematically for differantial diagnosis. We presented a very rare condition due to an metcloropramide. If the patient had not been evaluated well, the patient would have undergone to surgery for appendectomy.

## Conclusion

A systemic evaluation of the patient with acute right abdominal quadrant pain should be routine. Differential diagnosis for right abdominal quadrant pain have to be made. For this purpose we may use computerized tomography. In some instances such as acute nontravmatic torticollis or acute dystonic reactions, review of the initial clinical examination may be necessary to manage concomitant clinical findings or problems.
